# Predictors and outcomes of patient safety culture at King Abdulaziz Medical City, Jeddah, Saudi Arabia. A nursing perspective

**DOI:** 10.1186/s12912-023-01391-w

**Published:** 2023-07-03

**Authors:** Hawazen Rawas, Ebtsam Aly Abou Hashish

**Affiliations:** 1 College of Nursing, King Saud Bin Abdulaziz University for Health Sciences, Jeddah, Saudi Arabia; 2 King Abdullah International Medical Research Center, Jeddah, Saudi Arabia; 3 Ministry of the National Guard - Health Affairs, Jeddah, Saudi Arabia; 4 Medical/Surgical Nursing, College of Nursing-Jeddah, King Saud Bin Abdulaziz University for Health Sciences, King Abdulaziz Medical City, National Guard Health Affairs, P.O.Box. 9515, Mail Code 6565, Jeddah, 21423 Kingdom of Saudi Arabia; 5 Professor, Faculty of Nursing, Alexandria University, Alexandria, Egypt

**Keywords:** Nurses, Nursing perspective, Patient safety, Patient safety culture, Saudi Arabia

## Abstract

**Background:**

Patient safety culture assessment is viewed as the starting point from which action planning begins and helps hospitals get a good idea of the patient safety features that need immediate attention, identify the strengths and weaknesses of their safety culture, help units find their most common patient safety problems, and compare their scores to those of other hospitals. This study aimed to assess nurses’ perceptions of patient safety culture composites in a Saudi hospital in the Western region and to explore the association between patient safety culture predictors and outcomes, taking into consideration nurses' characteristics.

**Methods:**

This study employed a cross-sectional descriptive design with a convenience sample of 184 nurses who are working at inpatient care units at King Khaled Hospital- King Abdulaziz Medical City in Jeddah, Western region, Saudi Arabia. The data were collected through a structured questionnaire consisting of nurses’ demographics and work characteristics, and the Patient Safety Culture Hospital Questionnaire (HSOPSC), which proved valid and reliable. Descriptive status, correlation, and regression analysis were applied to patient safety culture composites for statistical analysis.

**Results:**

The overall positive response rate of the predictors of patient safety culture in the HSOPSC survey was 63.46%. The mean percent score for predictors ranged from 39.06% to 82.95%. "Teamwork within units" (82.95%) was the highest mean, followed by "organizational learning" (81.88%) and "feedback and communication about errors" (81.25%). In addition to the overall perceived patient safety (59.0%), safety grade, frequency, and number of events are also reported as safety outcome measures.

**Conclusions and recommendations:**

Regardless of the percentage of the safety culture domains, this study agrees that all the domains should be considered high-priority and focused areas for continuous improvement. The results confirmed the need for continuous staff safety training programs to improve their perception and performance of the safety culture.

## Background

Today, delivering safer care in complex, pressurized, and fast-moving environments is one of the greatest challenges facing health care [1]. Patient safety is considered a matter of great importance as an essential requirement in the hospital's accreditation [2–4]. Patient safety is identified as the prevention of damage to patients and emphasizes the prevention of errors, deriving lessons from mistakes, and the establishment of the healthcare delivery system based on a security culture that includes health professionals, organizations, and hospitals [1]. Improvement of patient safety with regard to the risks and consequences in a healthcare system depends on developing a patient safety culture [5]. Therefore, health institutions have constantly been working on developing measures to foster a patient safety culture [3, 4].

Patient safety culture refers to the perceived necessity of values, attitudes, skills, and behaviors, and it focuses on an institution’s patient care processes and related workforce [6]. Safety culture is determined by the degree to which individuals in the organization commit to safety, their determination to pursue safety in the midst of obstacles, and their willingness to report near-misses and adverse events. It also reflects the ability of both individuals and the organization to deal with risks and hazards, avoid errors, and achieve their goals [7]. Cultivating a safety culture is a fundamental component of many attempts to improve patient safety and quality of care [4].

### Safety culture in Saudi Arabia

Likewise, patient safety is an important issue in the Saudi health care context. In recent years, sensitivity to patient safety, medical errors, and clinical guidelines has increased among nurses, physicians, and other healthcare workers [8]. Patient safety and the initiative to develop safety cultures to assure patients’ freedom from harm have become central concerns in quality improvement in the healthcare system [2, 3]. Although the patient safety issue has become a major academic and public concern in the Saudi healthcare industry, patient safety has been the primary consideration on Saudi Arabia’s agenda for health policy [9]. Saudi Arabia has 13 administrative regions, and some studies have been done on the assessment of safety culture in the different Saudi regions. In Riyadh, for example, a study was conducted in 2015 to measure the patient safety culture in a multi-site medical city, and the results were compared to an earlier assessment conducted in 2012 [10]. The study reported that although there is significant progress in some areas, like teamwork within units, there are still areas for improvement in hospitals [10]. In the Hail region, the assessment results of the perceptions of 255 healthcare professionals toward patient safety culture from four major hospitals found a positive perspective toward patient safety culture aspects, with patient safety grade receiving the highest mean value, whereas handoffs and transitions received the least consensus [3]. The study also found a positive correlation between the patient safety dimensions and the participants’ characteristics. In addition, in Almadinah Almunawwarah, a study conducted to assess the patient safety culture as a quality indicator for a safe health system referred to the fact that none of the patient safety culture aspects achieved a positive score of 75% or more as an area of strength [4]. There is a suggestion to improve and promote applicable policies to improve the culture of patient safety in hospitals [4]. These studies indicated that a patient safety culture exists in Saudi Arabia, and somehow healthcare professionals continue to experience challenges in ensuring the safety of patients in hospitals based on patient safety standards. These studies recommend further research to continuously appraise the significance of patient safety culture and healthcare-based quality indicators. Therefore, this study aimed to extend the safety culture assessment research in the Western region of Saudi Arabia.

## Conceptual framework

According to the standard guidelines for monitoring patient safety culture in hospitals, the Agency for Healthcare Research and Quality (AHRQ) developed 12 basic composites that are considered basic elements of safety culture assessment in any hospital and were used as the current study framework, namely: communication openness, feedback and communication, frequency of events reported, handoffs and transitions, management support, nonpunitive response to error, organizational learning, overall perceptions of patient safety, staffing, supervisor/manager expectations and actions, teamwork across units, and teamwork within units [11].

Based on the framework, predictors, and outcomes of a positive patient safety culture in healthcare organizations, specifically hospitals, are identified in the literature. Predictors can include communication founded on mutual trust, good information flow, shared perception of the importance of safety, organizational learning, commitment from management and leadership, and the presence of a non-punitive approach to incident and error reporting. Patient safety culture outcomes can include the staff members’ perception of safety, the willingness of staff members to report events, the number of events reported, and an overall patient safety grade given by staff members to their units [12].

## Significant of the study

Although several studies were conducted in Saudi Arabia to assess the patient safety culture, there is still a gap in the literature regarding perceptions of the patient safety culture from a nursing perspective, especially in the Western region of Saudi healthcare [13, 14]. Also, limited evidence still exists about the linkage between predictors and outcomes of patient safety culture [12]. Thus, this study aims to contribute to the literature about safety culture in the Saudi healthcare context and to assess nurses’ perceptions of patient safety culture composites in a Saudi hospital in the Western region. Further explore the association between patient safety culture predictors and outcomes, taking into consideration nurses' characteristics. This study targets nurses as the primary care providers because their perspective may provide the best reflection of the existing patient safety culture. Nurses are uniquely positioned as frontline caregivers with opportunities to help develop a supportive unit environment for improving patient safety and reducing medical errors [3, 15].

The findings from this study might provide a description of the current status of patient safety in a representative tertiary hospital in the Saudi Western region, from the nurses’ perspective and the nurses’ approach to promoting a safety culture. Fostering a greater understanding of nurses’ perceptions and factors influencing them not only provided a baseline from which to work but also helped raise safety awareness, identify areas most in need of improvement, and target efforts to improve patient safety and decrease medical errors [3, 15, 16].

Likewise, this study might have a significant contribution and provide improved data to hospital and nursing administrators, chief nursing officers, performance improvement directors, and risk managers in developing, implementing, and evaluating training initiatives. As nurses’ perceptions and approaches of the existing safety culture and its impact on patient safety level are very important to the endurance of healthcare organizations, not only in understanding the attitude of nurses but in providing safer care [16].

## Methods

### Design

A cross-sectional descriptive design was employed in this study between May and July 2022.

### Sample and setting

Participants for this study included a convenience sample of nurses working in inpatient care units of King Khalid Hospital at King Abdulaziz Medical City (KAMC)-Jeddah, which is affiliated with the Ministry of National Guard Health Affairs (NHGA), Western region, Saudi Arabia. It is a 750-bed tertiary care hospital accredited by the Joint Commission International (JCI) in 2006. The inclusion criteria for the sample selection are being registered nurse with at least 6 months’ working experience in the hospital, being available at the time of data collection, and agreeing to participate. Nurse interns and students were excluded from the study. The sample size was being determined using Raosoft sample size calculator criteria: population size of 350, margin error of 5, confidence interval of 95%, significance level of.05, and thus, the minimum recommended sample size is 184.

### Instruments and measures

The present study used the original HSOPSC questionnaire in its English form, as the main language between all the health care providers in the hospital is English. In addition, some of the nurses are non-Arabic speakers and not from Arab countries.

The data were collected through a structured questionnaire that consists of two parts:

- Part 1: Nurses’ demographics and work characteristics such as age, sex, nationality, educational level, working unit, working experience, and hours worked per week.
- Part 2: The Hospital Patient Safety Culture Questionnaire (HSOPSC), developed by the Agency for Healthcare Research and Quality, was used to measure patient safety culture perceptions [11]. This questionnaire consists of 42 items and 12 composites as sub-dimensions, namely communication openness (3 items), feedback and communication (3 items), frequency of events reported (3 items), handoffs and transitions (4 items), management support (4 items), no punitive response to error (3 items), organizational learning (3 items), staffing (4 items), supervisor/manager expectations and actions (4 items), teamwork across units (4 items), teamwork within units (3 items), and overall perceptions of patient safety (4 items). The items were rated on a 5-point Likert scale in terms of agreement (strongly agree (5) to strongly disagree (1)), or frequency (always (5), to never (1)). The negative statements were reverse-coded. In addition to the mean score, which ranged between 1 and 5, the percent positive scores for the 12 patient safety culture composites are calculated by taking the average of the percent positive scores for the 3 or 4 items that make up the composite. Each percent positive score is a number between 0 and 100%, and the mean percent score was presented.

In addition, the survey also includes two questions that ask respondents to provide an overall grade on patient safety for their work area or unit and to indicate the number of events they reported over the past 12 months. These two questions are considered outcome variables in addition to the frequency of events reported and the overall perception of safety. Hence, four composites of patient safety culture are considered outcome variables, including the frequency of events reported, overall perception of safety, the patient safety grade, and the number of events reported; the rest are considered predictors.

#### Validity and reliability

The tool was subjected to content validity testing by the researchers and expert academic members. They were asked to evaluate the HSOPSC instrument based on item relevance, comprehensiveness, and comprehension. The content validity of it was evaluated using an index based on the rating agreement of the five experts, and the content validity index (CVI) was 85.30, proving that it is valid as CVI score above 0.79 is considered appropriate, between 0.79 and 0.70 is questionable and needs to be corrected and revised, less than 0.70 is unacceptable and should be removed [17]. In addition, a pilot study was conducted on 20 nurses to ensure its face validity, clarity, and applicability and estimate the time required to complete the study questionnaire. Accordingly, no modification was made. For reliability, the internal consistency coefficient for the HSOPSC was reported as 0.97 in a previous study [3], and in the current study it was 0.93.

### Data collection

After obtaining the institutional review board (IRB) approval of King Abdullah International Medical Research Center (KAIMRC), the hospital’s nurse director’s approval letter, and the unit's nurse managers’ permission, data was collected from nurses who agreed to participate in the study through the structured questionnaire. All nurses were invited by the researchers and informed of the purpose and design of the study and that their participation was completely voluntary. Participants were provided with informed written consent. All ethical considerations were maintained. Data were collected between May and July 2022, and the questionnaire required 25 min to be completed.

### Statistical analysis

Data were coded by the researchers and statistically analyzed using Statistical Package for the Social Science (25). Cronbach’s alpha correlation coefficient was used to test the study’s tool for internal reliability. Frequency and percentages were used to describe demographic characteristics. Descriptive statistics such as mean and standard deviation were applied to summarize the data. The student’s t test and Analysis of Variance (ANOVA) used to test the perceived difference in overall safety culture mean in relation to nurses’ demographic and work characteristics. To identify the significant predictors and outcome variables, correlation and regression analyses were applied to patient safety culture composites. *P* value of ≤ 0.05 is considered the level of significance.

## Results

### Socio-demographic characteristics

Socio-demographic characteristics are presented in Table 1. A total of 184 nurses participated in this study. Most participants were female nurses and non-Saudi nurses. Nearly half of them (46.7%) were aged between 31 and 40 years. The majority of nurses (90.8%) held a baccalaureate degree. Nurses were distributed among the different units. The highest percentage of nurses (44.6%) had 1–5 years of experience, and about two-thirds of nurses (67.4%) worked an average of 40–59 h per week. See Table 1 for more values.

**Table 1:** Demographic characteristics of the studied nurses (*N* = 184)

**Demographic** characteristics	**No.**	**%**
**Sex**		
Male	32	17.4
Female	152	82.6
**Nationality**		
Saudi	35	19.0
Non-Saudi	149	81.0
**Age category**		
20—< 30 years	71	38.6
30—< 40 years	86	46.7
40—< 50 years	22	12.0
≥ 50 years	5	2.7
**Education**		
Bachelor level	167	90.8
Institute diploma	14	7.6
Master	3	1.6
**Primary work area**		
Medical	52	28.3
Surgical	52	28.3
Obstetrics	12	6.5
Pediatrics	15	8.2
Oncology	9	4.9
ICU	44	23.9
**Years of experience**		
less than 1 year	23	12.5
1 to 5 years	82	44.6
6 to 10 years	45	24.5
11 to 15 years	24	13.0
16 to 20 years	6	3.3
21 years or more	4	2.2
**Working hours per week**		
20 to 39 h per week	10	5.4
40 to 59 h per week	124	67.4
60 to 79 h per week	50	27.1

### Perceived predictors of patient safety culture

Tables 2 and 3 show the mean and mean percent scores of the predictors and outcomes of patient safety culture as measured on the 12 HSOPSC dimensions. Regarding the perceived predictors of patient safety, Table 2 reveals the overall PSC predictors' mean was 63.46%, and the mean percent score ranged from 39.06% to 82.95% for the predictors. "Teamwork within units" (82.95%) was the highest mean, followed by "organizational learning" (81.88%) and "feedback and communication about errors" (81.25%). While there were three weak dimensions that were less than 50%, "handoffs and transitions" (39.06%) was the lowest, followed by staffing (40.08%) and nonpunitive response to errors (43.89%).

**Table 2:** Perceived predictors of patient safety culture in the studied hospital

Predictors of patient safety culture	Mean score ± SD	Mean % Score ± SD
1. Teamwork within units	4.32 ± 0.52	82.95 ± 13.02
2. Organizational learning—continuous improvement	4.28 ± 0.57	81.88 ± 14.13
3. Staffing	2.60 ± 0.56	40.08 ± 13.97
4. Nonpunitive Response to Errors	2.76 ± 0.77	43.89 ± 19.17
5. Supervisor/manager expectations & actions promoting patient safety	3.91 ± 0.69	72.86 ± 17.38
6. Management Support for Patient Safety	3.80 ± 0.72	70.15 ± 18.05
7. Teamwork across units	3.69 ± 0.76	67.29 ± 19.08
8. Handoffs & transitions	2.56 ± 0.94	39.06 ± 23.44
9. Feedback & communication about error	4.25 ± 0.74	81.25 ± 18.57
10. Communication openness	3.23 ± 0.97	55.80 ± 24.28
**Overall**	**3.54 ± 0.36**	**63.46 ± 9.02**

*SD* Standard deviation

**Table 3:** Perceived four outcomes of patient safety culture in the studied hospital

Outcome of Patient safety culture	Mean score ± SD	Mean % Score ± SD
Overall Perceptions of Patient Safety	3.36 ± 0.61	59.00 ± 15.16
Frequency of Events Reported	3.95 ± 0.71	73.82 ± 17.98
**Patient Safety Grade**	**No.**	**%**
Excellent	35	19.0
Very Good	56	30.4
Acceptable	91	49.5
Poor	2	1.1
**Average Patient Safety Grade**	2.67 ± 0.79	
**Number of Events Reported**	**No.**	**%**
No event reports	81	44.0
1 to 2 event reports	60	32.6
3 to 5 event reports	25	13.6
6 to 10 event reports	18	9.8
**Average of Events Reported**	1.89 ± 0.98	

*SD* Standard deviation

### Perceived outcomes of patient safety culture

Table 3 illustrates the four outcomes of patient safety culture, including "overall perceptions of safety, frequency of events reported, patient safety grade, and number of events reported". The mean percent score for the overall perception of patient safety was 59%. About half of nurses (49.5%) rated the patient safety grade as acceptable. The mean score for the frequency of events reported was 73.82%. The majority of nurses reported that most of the time and always they report mistakes, which are caught and corrected before affecting the patient, have no potential to harm the patient, or could harm the patient but did not. About one third of nurses (32.6%) reported only one or two events in the past 12 months, while 44% indicated that they had not reported any events.

### Correlation and regression analysis of patient safety culture predictors and outcomes

The four outcome variables were tested for correlation and regressed against the 10 predictor composite scores as shown in Tables 4 and 5. Table 4 shows a significant positive correlation between all predictors of patient safety and the frequency of events reported (*r* = 0.147–057, *p* < 0.05), except for nonpunitive responses to errors and communication openness. While handoffs and transitions show a negative correlation with the frequency of events reported (*r* = -0.153, *p* = 0.038). In addition, the regression analysis in Table 5 reveals that these predictors together can contribute 42.9% to the variance in the frequency of events reported (*R*^2^ = 0.429, *p* < 0.001). Management support for patient safety, teamwork across units, feedback, and communication about errors play a significant role in this prediction.

**Table 4:** Correlation and regression analysis of patient safety culture predictors and outcomes

Predictors of patient safety culture	Outcomes of patient safety culture							
	Frequency of Events Reported		Patient Safety Grade		Number of Events Reported		**Overall Perceptions of Patient Safety**	
	**r**	**p**	**r**_**s**_	**P**	**r**_**s**_	**p**	**r**	**p**
1. Teamwork within units	0.349	< 0.001^*^	0.239	0.001^*^	-0.063	0.394	0.393	< 0.001^*^
2. Organizational learning—continuous improvement	0.380	< 0.001^*^	0.150	0.042^*^	-0.162	0.028^*^	0.432	< 0.001^*^
3. Staffing	0.147	0.047^*^	0.019	0.796	-0.163^*^	0.027^*^	0.397	< 0.001^*^
4. Nonpunitive response to errors	-0.089	0.228	0.142	0.054	0.045	0.544	0.547	< 0.001^*^
5. Supervisor/manager expectations & actions promoting patient safety	0.301	< 0.001^*^	0.287	< 0.001^*^	-0.194	0.008^*^	0.531	< 0.001^*^
6. Management support for patient safety	0.400	< 0.001^*^	0.346	< 0.001^*^	-0.198	0.007^*^	0.471	< 0.001^*^
7. Teamwork across units	0.204	0.005^*^	0.460	< 0.001^*^	-0.315	< 0.001^*^	0.583	< 0.001^*^
8. Handoffs & transitions	-0.153	0.038^*^	-0.402	< 0.001^*^	0.320	< 0.001^*^	0.439	< 0.001^*^
9. Feedback & communication about error	0.579	< 0.001^*^	0.210	0.004^*^	-0.239	0.001^*^	0.382	< 0.001^*^
10. Communication openness	0.076	0.307	0.548	< 0.001^*^	-0.315	< 0.001^*^	0.450	< 0.001^*^

r: Pearson coefficient, r_s_: Spearman coefficient. *: Statistically significant at *p* ≤ 0.05

**Table 5:** Multivariate analysis Logistic regression for outcomes

Predictors	Frequency of Events Reported					Patient Safety Grade					Number of Events Reported					Overall Perceptions of Patient Safety				
	**B**	**Beta**	**P**	**95% CI**		**B**	**Beta**	**P**	**95% CI**		**B**	**Beta**	**P**	**95% CI**		**B**	**Beta**	**P**	**95% CI**	
				**LL**	**UL**				**LL**	**UL**				**LL**	**UL**				**LL**	**UL**
Teamwork within units	0.087	0.063	0.446	-0.137	0.310	0.006	0.096	0.223	-0.004	0.015	0.006	0.085	0.386	-0.008	0.021	0.073	0.063	0.360	-0.084	0.230
Organizational learning—continuous improvement	0.000	0.000	0.999	-0.222	0.222	-0.009	-0.163	0.056	-0.018	0.000	-0.003	-0.042	0.687	-0.017	0.011	0.231	0.215	0.004^*^	0.075	0.387
Staffing	-0.017	-0.013	0.860	-0.202	0.169	-0.011	-0.188	0.008^*^	-0.018	-0.003	-0.008	-0.110	0.206	-0.020	0.004	0.075	0.069	0.254	-0.055	0.206
Nonpunitive response to errors	-0.112	-0.119	0.087	-0.240	0.016	0.001	0.034	0.609	-0.004	0.007	0.010	0.192	0.021^*^	0.002	0.018	0.323	0.408	< 0.001^*^	0.233	0.414
Supervisor/manager expectations & actions promoting patient safety	0.122	0.117	0.239	-0.081	0.324	0.001	0.025	0.794	-0.007	0.010	0.001	0.020	0.866	-0.012	0.014	0.045	0.052	0.531	-0.097	0.188
Management support for patient safety	0.370	0.371	< 0.001^*^	0.173	0.568	0.002	0.047	0.626	-0.006	0.010	0.009	0.169	0.159	-0.004	0.022	-0.093	-0.110	0.190	-0.231	0.046
Teamwork across units	-0.337	-0.358	0.001^*^	-0.532	-0.142	0.004	0.102	0.309	-0.004	0.012	-0.005	-0.090	0.473	-0.017	0.008	0.258	0.325	0.000^*^	0.121	0.395
Handoffs & transitions	0.074	0.097	0.240	-0.050	0.198	-0.011	-0.323	< 0.001^*^	-0.016	-0.006	0.015	0.361	< 0.001^*^	0.007	0.023	0.004	0.006	0.929	-0.083	0.091
Feedback & communication about error	0.517	0.534	< 0.001^*^	0.349	0.684	-0.002	-0.055	0.514	-0.009	0.005	-0.002	-0.031	0.769	-0.012	0.009	-0.030	-0.037	0.614	-0.148	0.088
Communication openness	-0.061	-0.082	0.215	-0.158	0.036	0.015	0.473	< 0.001^*^	0.011	0.019	-0.008	-0.209	0.009^*^	-0.015	-0.002	0.120	0.192	0.001^*^	0.052	0.188
	***R***^**2**^ = 0.429,*F* = 13.020^*^,*p* < 0.001^*^					***R***^**2**^ = 0.478,*F* = 15.810^*^,*p* < 0.001^*^					***R***^**2**^ = 0.195,*F* = 4.182^*^,*p* < 0.001^*^					***R***^**2**^ = 0.603,*F* = 26.311^*^,*p* < 0.001^*^				

F, p: f and p values for the model, R^2^: Coefficient of determination, B: Unstandardized Coefficients, Beta: Standardized Coefficients

^*^ Statistically significant at *p* ≤ 0.05

Patient safety grade shows a significant positive correlation with all predictors of patient safety (rs = 0.150—0.548, *p* < 0.05) except for staffing and nonpunitive response to errors, while handoffs and transitions show a negative correlation with patient safety grade (rs = -0.402, *p* < 0.001). In addition, the regression analysis reveals that these predictors together can contribute to 47.8% of the variance of patient safety grade (*R*^2^ = 0.478, *p* < 0.001). Staffing, handoffs and transitions, and communication about errors play a significant role in this prediction.

On the other hand, Table 4 shows a significant negative correlation between all predictors of patient safety and the number of events reported (rs = -0.162 – 0.548, *p* < 0.05), except for teamwork within units and nonpunitive responses to errors. While handoffs and transitions show a positive correlation with the number of events reported (rs = 0.320, *p* < 0.001). In addition, the regression analysis reveals that these predictors together can contribute 19.5% in the variance of frequency of events reported (*R*^2^ = 0.195, *p* < 0.001). Nonpunitive responses to errors, handoffs and transitions, and communication openness play a significant role in this prediction.

Moreover, overall perceptions of patient safety show a significant positive correlation with all predictors of patient safety (*p* < 0.001). The regression analysis reveals that these predictors together can contribute to 60.3% of the variance of overall perceptions of patient safety (*R*^2^ = 0.603, *p* < 0.001). Organizational learning—constant improvement, nonpunitive response to errors, cross-functional teamwork, and communication openness—plays a significant role in this prediction. For more values on correlation and regression analysis, see Table 4.

It seems that communication openness, feedback, and communication about errors, non-punitive response to errors, handoffs and transitions, and teamwork were the most frequently composites that could contribute to or predict the safety culture outcomes in the hospital.

### Nurses’ demographics characteristics and overall patient safety culture

The result revealed that there is no significant difference in perceived patient safety culture according to nurses' socio-demographic characteristics, including nationality (Saudi and non-Saudi), sex, age category, educational level, or length of years of experience. Only the work unit and hours per week showed significant differences in the overall patient safety culture, where nurses working in pediatric units had a higher mean score than the other units (*F* = 25.865, *p* < 0.001). Furthermore, nurses who worked an average of 40 to 59 h per week had a higher patient safety culture mean than nurses who worked less or more than this average (*F* = 11.906, *p* < 0.001). See Table 6.

**Table 6:** Differences in perceived overall patient safety culture according to nurses’ demographics

Nurses’ Demographics	Overall patient safety culture	Test of sig.	*p*
	**Mean ± SD**		
**Nationality**			
Saudi	3.46 ± 0.33	t = 1.348	0.179
Non-Saudi	3.56 ± 0.37		
**Sex**			
Male	3.59 ± 0.33	t = 1.031	0.304
Female	3.53 ± 0.37		
**Age category**			
20—< 30 years	3.58 ± 0.34	*F* = 0.628	0.535
30—< 40 years	3.54 ± 0.37		
40—< 50 years	3.40 ± 0.37		
≥ 50 years	3.53 ± 0.34		
**Education**			
Bachelor level	3.54 ± 0.36	*F* = 0.628	0.535
Institute diploma	3.45 ± 0.32		
Master	3.65 ± 0.36		
**Primary work area (unit)**			
Medical	3.44 ± 0.27	25.865^*^	< 0.001^*^
Surgical	3.59 ± 0.30		
Obstetrics	3.52 ± 0.21		
Pediatrics	3.96 ± 0.23		
Oncology	3.04 ± 0.12		
ICU	3.78 ± 0.31		
**Years of experience**			
less than 1 year	3.51 ± 0.28	*F* = 2.093	0.068
1 to 5 years	3.52 ± 0.39		
6 to 10 years	3.63 ± 0.37		
11 to 15 years	3.58 ± 0.24		
16 to 20 years	3.23 ± 0.37		
21 years or more	3.28 ± 0.32		
**Work hours per week**			
20 to 39 h per week	3.37 ± 0.18	*F* = 11.906^*^	< 0.001^*^
40 to 59 h per week	3.64 ± 0.33		
60 to 79 h per week	3.32 ± 0.35		

F: F for One way ANOVA test, t: Student t-test, *Statistically significant at *p* ≤ 0.05

## Discussion

As patient safety is a fundamental component of healthcare quality, the Patient Safety Culture (PSC) assessment provides an organization with insight into its employees' perceptions and attitudes toward patient safety, as well as the strategies required to improve performance, rather than blaming culture. This study aimed to assess the patient safety culture from the nurses’ perspective at KAMC, in the Western region of Saudi Arabia. The discussion will present the perceived level and outcomes of patient safety culture and how they will be explained by the predictors of patient safety in the light of available studies done in Saudi healthcare settings.

### Perceived level and outcome of patient safety culture

Generally, the present study revealed that the overall positive response rate of the predictors of patient safety culture in the HSOPSC survey was 63.46%, which indicates an average level of patient safety culture in the inpatient care units at the KAMC. Also, about half of the nurses rated overall patient safety at KAMC as "excellent" or "very good," and the other half rated it as "acceptable". Although nurses realize the importance of prioritizing patient safety as a determinant of quality health services in hospitals, this perceived average in the current hospital could be related to many factors that affect patient safety, such as staffing and the COVID-19 situation in the hospital, especially during 2020–2021. Compared to the previous studies in Saudi Arabia, the observed positive response rate in this study is comparable to those reported in AlSabaani in the Southern region of Saudi Arabia [18], while the result is lower than that in Aboufour and Subbarayalu’s study [19], which reported an average of 67.12% in hospitals in the Eastern region. The variation in the reported average and positive patient safety culture could be attributed to the different hospitals' structures, the organization’s awareness of patient safety culture, hospital experience, size, function, types of services, and samples included in each study [20, 21]. This finding suggests that hospital management could consider implementing strategies aiming to teach and promote supervisory behaviors that encourage the nursing staff to report information regarding safety and participate in safety initiatives.

Moreover, the four outcomes of patient safety culture reported in this study are "overall perceptions of safety, frequency of events reported, patient safety grade, and number of events reported". The mean percent score for the overall perception of patient safety was 59% and 73.82% for the frequency of events reported. The majority of nurses reported that most of the time and always report mistakes, which are caught and corrected before affecting the patient, have no potential to harm the patient, or could harm the patient, but did not and about one-third of nurses (32.6%) reported only one or two events in the past 12 months, while 44% indicated that they had not reported any events. Error reporting was reported in many studies among the factors that affect patient safety. Although nurses in the current study show willingness to report errors frequently but might be afraid and worried about the punitive response to error reporting. This justification could also be supported by the below average given to nonpunitive response to errors (43.89 %) as a predictor to patient safety. The following discussion of different predictors of patient safety will explain the reasons of these outcomes.

### Predictors of patient safety culture (strengths and weakness)

The results of the study identified that patient safety culture is generally accepted and has strengths, particularly in the aspects of "teamwork within units, organizational learning and continuous improvement, and feedback and communication about errors." On the other hand, the three safety culture domains that have potential for improvement are "staffing, handoff, and transition communication" and "non-punitive response," which had positive responses that were less than 50%. These areas might be considered weaknesses in the patient safety culture in the current hospital that require interventions and should be prioritized and improved to promote patient safety. These findings match to some extent what was reported in previous studies that were conducted among Saudi hospitals [18, 19, 22, 23]. For instance, Alquwez et al. [22] conducted a study in three Saudi hospitals and found that among the 12 composites of the HSOPSC, nurses perceived only teamwork within units and organizational learning—continuous improvement—as strengths in patient safety areas [22]. While the overall perception of patient safety, handoffs and transitions, communication openness, staffing, the frequency of events reported, and nonpunitive responses to errors were identified as weaknesses.

Another study done by El-Jardali et al. (2014) assessed the patient safety culture of a large teaching hospital in Riyadh region and identified that organizational learning, continuous improvement, and teamwork within units are the strengths of the hospital, whereas nonpunitive response to error, staffing, and communication openness are its weaknesses [21]. Also, it has been found that handoffs and transitions, staffing, the frequency of events reported, and nonpunitive responses to errors are frequently reported as areas of weakness [9]. However, Alrowely & Ghazi Baker [24] found that the dimensions "staffing" and "nonpunitive response to error" had a positive response score. The present study’s results suggest that the areas of handovers and transferring patients’ information across hospital units and during shift changes within the KAMC need significant concern and improvement.

Among the different predictors, *teamwork* within units had the highest positive composite score and was considered an area of strength in this study, which reflects that the nurses within the unit at KAMC support and treat each other with respect, and there is a cooperative working atmosphere. This finding is consistent with the results of previous studies, which revealed that "teamwork within units" is a highly rated patient safety dimension by the nurses in Saudi Arabia [14, 25]. In contrast, another study recorded "teamwork within units" with the lowest positive response score of 21.4% [23]. In this regard, Albalawi, Kidd, and Cowey [26] reported that the variation in the results of previous studies could be related to the different policies and procedures for the communication system within Saudi organizations. In addition to the leadership style of nurse managers and the extent to which they support team spirit in the unit [27]. In this regard, we emphasize supportive leadership, teamwork, and professional communication training as an effective strategy for improving safety culture.

The other patient safety culture dimensions that recorded positive response rates in the present study were "*organizational learning, continuous improvement, feedback, and communication about errors,"* which implied that KAMC inspires workforces to learn and develop themselves through open channels of communication and error reporting. The results might be attributable to the nature of KAMC as a teaching hospital accredited by JCI and affiliated with King Saud bin Abdul-Aziz University for Health Sciences (KSAU-HS), which entails a learning environment where staff and students are willing to improve their knowledge and practices through conferences, workshops, and sharing their experiences to achieve safe and quality patient care. Likewise, the literature about Saudi Arabia's patient safety culture declares that organizational learning is a strength because government hospitals in Saudi Arabia have their own unit or department for continuing education that values and supports the educational support and learning of staff [28]. Additionally, nurses and other health care workers in Saudi Arabia should complete their continuing medical education hours to renew their licenses and continue working in a clinical setting [22]. Previous studies also revealed positive responses on these dimensions in Saudi hospitals [18, 19, 25, 29], while the observed scores are better than the outcomes reported by Aboufour and Subbarayalu [19].

In contrast, *staffing* is one of the significant challenges that face the nursing profession in Saudi Arabia and worldwide. About two-thirds of nurses (67.4%) in the current study worked an average of 40 to 59 h per week, which indicates a heavy workload on nurses and might lead to more errors that affect the quality of care and patient safety. Two Saudi studies came to similar conclusions [20, 22]. They said that insufficient staffing is a common problem that hurts nurses because of heavy workloads and long hours. This is also shown by the fact that nurses who work more than 40 h per week get a high score. When the number of personnel needed to provide patient care is less than ideal, most employees are overworked, burned out, stressed, and sleepless, which can lead to gaps in performance, affecting quality and patient outcomes. Alsayed, Abou Hashish, and Alshammari also mentioned the Saudi staff shortage as well as increased workload and demands on nurses, affect their performance and well-being as well as patient safety [30].

Moreover, the *Handoff and Transitions* score (39.06%) indicates that problems often occur in the exchange of information and ineffective communication inside or across hospital units, which consequently could adversely affect patient safety. The multicultural nature of nurses working in Saudi hospitals may jeopardize effective communication due to varying cultural beliefs, languages, religions, and nationalities. Consistent with these findings, some studies discovered that communication and language barriers at the health provider level, primarily between Saudi nurses and non-Saudi expatriate nurses, affect health services coordination and planning and introduce another risk to patient safety due to miscommunication [3, 31]. Also, it has been found that inadequate handover communication and documentation put patients at increased risk for adverse events because of delays in treatment or procedures [32].

Another challenge in this study is the composite of *nonpunitive responses to errors,* which could be due to a variety of factors. Also, about 44.0% of nurses in this study reported no incidence of errors or events in the past 12 months as an outcome measure in this study. Reporting errors seems to be a process that is often avoided, and efforts to identify mistakes may be undervalued. Nurses may still be afraid to report such incidents for fear of repercussions, or they may lack adequate encouragement from nurse managers to report errors in their work. Also, nurses might believe that they only need to report errors that occur, but they do not realize that it is also important to report potential errors. Similar justifications were reported by [9, 11, 33]. Compared to previous Saudi studies, the under-reported adverse events in the past 12 months are consistent with those reported by Alquwez et al. [22]. While the reported score was lower than that of a previous study, in the Alshammari et al. study, as 63.53% of the participants responded that they had never reported an event of patient safety [3]. However, Alrowely and Ghazi Baker observed that the "nonpunitive response to error" had a positive response score [24]. Also, these results are consistent with those of previous studies conducted among Saudi nurses, wherein the aspect of "nonpunitive response to error" needs improvement [3, 19, 22]. In this regard, Aljabari and Kadhim illustrated that within the same country, reported barriers differed from one facility to another [34]. Moreover, it has been revealed that the presence or lack of elements encouraging a positive safety culture, such as a blame and shame culture in dealing with adverse events, open communication, and management support, could explain the discrepancy in the overall perception of errors’ reporting [35].

The present study reveals that all measured predictors together can contribute to 60.3% of the variance in overall perceptions of patient safety culture. Also, the study reveals that patient safety predictors together can contribute to 47.8% of the variance of the patient safety grade, 42.9% of the variance of the frequency of events reported, and 19.5% of the variance of the frequency of events reported. It seems that "communication openness, feedback, and communication about errors, a non-punitive response to errors, handoffs and transitions, and teamwork" were the most frequently mentioned composites that played a significant role in this prediction and could contribute to or predict the safety culture outcomes in the hospital. The previous discussion of different predictors could explain these findings. In the present study, all aspects of communication and handover reported a significant effect on patient safety culture but needed more improvement. This result is consistent with Alshammari et al., who considered the overall patient safety grade for the hospital an outcome variable [3]. Also, El-Jardali et al. have found that the number of reported events filed in the studied Saudi hospital was significantly associated with composite questions measuring communication openness [21]. In addition, previous studies reported aspects such as "communication openness," "staff handoffs and transitions," and "a number of events reported" that had scope for further improvement [3, 36].

### Nurses’ demographic characteristics and overall patient safety culture

The results of the present study revealed that nurses' nationality (Saudi and non-Saudi), sex, age category, educational level, and working experience showed no significant difference in the overall perceived patient safety culture. On the other hand, significant differences were found in the perceived overall patient safety culture according to the work units and hours per week, where nurses working in paediatrics units had a higher mean score than the other units, and nurses who worked an average of 40 to 59 h per week had a higher patient safety culture mean than nurses who worked less or more than this average. This difference might be related to the complex nature of the work in pediatric units and the associated workload on nurses. In addition, previous researchers reported that prolonged exposure to patient care procedures provides healthcare professionals with more familiarity, awareness, and updated knowledge regarding patient safety and other types of work performed in the workplace [3, 22, 37], especially with vulnerable patients, such as paediatric ones.

In contrast, the demographics of nurses were identified as predictors of their perceptions of patient safety culture in other studies [12, 22, 38], where nationality, educational attainment, length of service, current position, and direct patient contact were significant predictors of the perceived patient safety culture of nurses [22], and non-Saudi nurses and less experienced nurses perceived patient safety culture more positively [12]. Therefore, patient safety culture measurements should consider the interaction between organizational and individual factors, which could provide a better understanding of group dynamics and individual attitudes toward patient safety culture.

#### Limitations of the study

Certain limitations are admitted in this study. First, the study used a sample restricted to one university hospital in the Western region of Saudi Arabia, which might limit the generalizability of the study. Second, the study utilized self-reported questionnaires, which might introduce bias into the results. Larger-scale studies involving more hospitals and data triangulation are required to form a comprehensive picture of the situation and create a database of patient safety culture in Saudi university hospitals. The current findings were purely based on the one-shot survey conducted. Following the given context, we suggest conducting a follow-up study on patient safety using a mixed-methods design. Nonetheless, the findings of the study contributed immensely to the literature on patient safety culture in the Western region of Saudi Arabia, especially in KAMC, which serves as one of the major teaching hospitals for nursing students in the Makkah and Western region.

## Conclusions

The results of this study showed that nurses had an acceptable impression of patient safety culture and nurses demonstrated a positive perception on many aspects of patient safety culture such as "teamwork within units, organizational learning and continuous improvement, and feedback and communication about errors". Despite that, King Khalid hospital-KAMC still has room for improvement in several areas. Nurses' general impressions of the emphasis placed on patient safety can be greatly influenced by the implementation of measures designed to boost the quality of care provided. This is notably true in the areas of improving the quality of handoff and information transition and communication, creating a nonpunitive culture, providing consistent education to nurses across all units, and providing adequate staffing which need more support and improvement.

## Recommendations

Investing in practices that strengthen patient safety is crucial if the hospital is to improve its overall performance and quality of services. The present study displays an average patient safety culture (PSC) in the majority of the domains. Regardless of the percentage of each domain, we agree that all the domains should be considered high-priority, focused areas for continuous improvement without regard to their individual characteristics and experiences. The study's findings recommend the creation of effective interventions to enhance patient safety procedures at King Khalid Hospital, which could also be applicable to other Saudi hospitals. Therefore, certain recommendations are suggested, as follows: The hospital and nursing administrators should continue to:

- Consider a systematic approach toward patient safety training, safety control, professional communication, and standardized handover to ensure that staff are sharing patients’ information during shift changes with colleagues. Nurses should play an active role in providing such educational interventions.
- Promote supportive leadership behaviors and a blame-free environment to support error reporting and proactive risk management that focuses on the errors in the system or process rather than the individual’s fault.
-Calculating workload, reallocating staff, rescheduling shifts, and distributing standardized communication forms as helpful and recommended strategies for promoting staff wellbeing, teamwork, and professional relationships among staff and reducing the burden on nurses.

In addition to what are recommended in the limitation section, future studies should investigate the effect of nurses' individual and organizational factors on the perceived patient safety culture. Also, we suggested analyzing and reporting the correlation between the participants' demographic characteristics and perceived predictors and outcomes of patient safety culture.

## Data Availability

All data generated or analyzed during this study are included in this published article [and its supplementary information files].

## References

[1] World Health Organization . Patient Safety: Making health care safer. 2017. https://apps.who.int/iris/bitstream/handle/10665/255507/WHO-HIS-SDS-2017.11-eng.pdf.

[2] World Health Organization (WHO). Patient safety. Global action on patient safety. 2019. https://apps.who.int/gb/ebwha/pdf_files/WHA72/A72_26-en.pdf.

[3] Alshammari F, Pasay-an E, Alboliteeh M, Alshammari MH, Susanto T, Villareal S, Indonto MC, Gonzales F. A survey of hospital healthcare professionals’ perceptions toward patient safety culture in Saudi Arabia. Int J Afr Nurs Sci. 2019;11:100149. 10.1016/j.ijans.2019.100149.

[4] Mahrous MS (2018). Patient safety culture as a quality indicator for a safe health system: experience from Almadinah Almunawwarah, KSA. J Taibah Univ Med Sci.

[5] Sonğur C, Özer Ö, Gün Ç, Top M (2018). Patient safety culture, evidence-based practice and performance in nursing. Syst Pract Action Res.

[6] Sendlhofer G, Wölfler C, Pregartner G (2015). Patient safety culture within a university hospital: feasibility trial. Saf Health.

[7] Sherwood G (2015). Perspectives: Nurses' expanding role in developing safety culture: quality and safety education for nurses–Competencies in action. J Res Nurs.

[8] Güneş ÜY, Gürlek Ö, Sönmez M (2016). A survey of the patient safety culture of hospital nurses in Turkey. Collegian.

[9] Elmontsri M, Almashrafi A, Banarsee R, Majeed A. Status of patient safety culture in Arab countries: a systematic review. BMJ Open. 2017;7(2):e013487.10.1136/bmjopen-2016-013487. PMID: 28237956. 10.1136/bmjopen-2016-013487 PMC5337746 28237956

[10] Alswat K, Abdalla RA, Titi MA, Bakash M, Mehmood F, Zubairi B, Jamal D, El-Jardali F (2017). Improving patient safety culture in Saudi Arabia (2012–2015): trending, improvement and benchmarking. BMC Health Serv Res.

[11] Agency for Healthcare Research and Quality AHRQ. Hospital Survey on Patient Safety Culture. 2015. http://www.ahrq.gov/professionals/qualitypatientsafety/patientsafetyculture/hospital/index.html. 21249886

[12] El-Jardali F, Dimassi H, Jamal D, Jaafar M, Hemadeh N (2011). Predictors and outcomes of patient safety culture in hospitals. BMC Health Serv Res.

[13] Abou Hashish EA, Aljuaid WA, Almuzaini O. Saudi nursing students attitudes towards patient safety and the influencing factors. A quantitative and qualitative study at the college of nursing-jeddah. Int J Nurs Educ Res. 2020;8(1):53–66. 10.5958/2454-2660.2020.00011.3.

[14] Aboshaiqah AE, Baker OG (2013). Assessment of nurses' perceptions of patient safety culture in a Saudi Arabia hospital. J Nurs Care Qual.

[15] Top M, Tekingündüz S (2015). Patient safety culture in a Turkish public hospital: a study of nurses’ perceptions about patient safety. Syst Pract Action Res.

[16] Alonazi NA, Alonazi AA, Saeed E, Mohamed S. The perception of safety culture among nurses in a tertiary hospital in Central Saudi Arabia. Sudanese J Paediatr. 2016;16(2):51. PMC5237835. PMC5237835 28096559

[17] Madadizadeh F, Bahariniya S. Tutorial on how to Calculating content validity of scales in medical research. Perioper Care Operat Room Manage. 2023:100315. 10.1016/j.pcorm.2023.100315.

[18] Alsabaani AA (2020). Perceptions of patient safety culture among physicians and nurses in a tertiary hospital in southwestern Saudi Arabia. Middle East J Fam Med.

[19] Aboufour MA, Subbarayalu AV. Perceptions of patient safety culture among healthcare professionals in Ministry of Health hospitals in Eastern Province of Saudi Arabia. Inf Med Unlocked. 2022;28:100858. 10.1016/j.imu.2022.100858.

[20] Ali Ali HM, Abdul-Aziz AM, Darwish EA, Swelem MS, Sultan EA (2022). Assessment of patient safety culture among the staff of the University Hospital for Gynecology and Obstetrics in Alexandria Egypt. J Egypt Public Health Assoc.

[21] El-Jardali F, Sheikh F, Garcia NA, Jamal D, Abdo A (2014). Patient safety culture in a large teaching hospital in Riyadh: baseline assessment, comparative analysis and opportunities for improvement. BMC Health Serv Res.

[22] Alquwez N, Cruz JP, Almoghairi AM, Al-otaibi RS, Almutairi KO, Alicante JG, Colet PC (2018). Nurses’ perceptions of patient safety culture in three hospitals in Saudi Arabia. J Nurs Scholarsh.

[23] Aljaffary A, Al Yaqoub F, Al Madani R, Aldossary H, Alumran A. Patient safety culture in a teaching hospital in Eastern Province of Saudi Arabia: assessment and opportunities for improvement. Risk Manage Healthc Policy. 2021:3783–95. 10.2147/RMHP.S313368. 10.2147/RMHP.S313368 PMC8447945 34548827

[24] Alrowely Z, Ghazi Baker O. Assessing building blocks for patient safety culture—a quantitative assessment of Saudi Arabia. Risk Manage Healthc Policy. 2019:275–85. 10.2147/RMHP.S223097. 10.2147/RMHP.S223097 PMC6902844 31827340

[25] Hazazi MA, Qattan AM (2021). Exploring strength areas of patient safety culture improvement in KAMC, Makkah Saudi Arabia. Am J Nurs.

[26] Albalawi A, Kidd L, Cowey E. Factors contributing to the patient safety culture in Saudi Arabia: a systematic review. BMJ Open. 2020;10(10):e037875. 10.1136/bmjopen-2020-037875. 10.1136/bmjopen-2020-037875 PMC7559108 33055115

[27] Abou Hashish E, Farghaly S. Exploring how nurse managers’ knowledge of succession planning affects their leadership and organisational resilience. Nurs Manag. 2021;28(6). 10.7748/nm.2021.2006. 10.7748/nm.2021.2006 35486497

[28] Alkhazim MA, Althubaiti A, Al-Ateeg H, Alkhwaiter M, AlNasser MM (2015). Delivering effective continuous medical education in Saudi Arabia: some critical issues. Health Prof Educ.

[29] Alrasheadi BA, Alamri MS, Aljohani KA, Al-Dossary R, Albaqawi H, Alharbi J, Hosis KA, Aljohani MS, Almadani N, Falatah R, Alotaibi JS (2022). Nurses’ perception of safety culture in medical− surgical units in hospitals in Saudi Arabia. Medicina.

[30] Alsayed SA, Abou Hashish EA, Alshammari F (2022). Occupational fatigue and associated factors among saudi nurses working 8-hour shifts at public hospitals. SAGE Open Nurs.

[31] Alshammari M, Duff J, Guilhermino M (2019). Barriers to nurse–patient communication in Saudi Arabia: an integrative review. BMC Nurs.

[32] Manias E, Geddes F, Watson B, Jones D, Della P (2016). Perspectives of clinical handover processes: a multi-site survey across different health professionals. J Clin Nurs.

[33] Abou Hashish EA, El-Bialy GG (2013). Nurses’ perceptions of safety climate and barriers to report medication errors. Life Sci J.

[34] Aljabari S, Kadhim Z. Common barriers to reporting medical errors. Scientific World J. 2021;2021. 10.1155/2021/6494889. 10.1155/2021/6494889 PMC8211515 34220366

[35] El Shabrawy E, Anwar M. Assessment of patient safety culture among health care workers in Beni-Suef University Hospital Egypt. Egypt J Community Med. 2017;35(3):11–9. 10.21608/ejcm.2017.4089.

[36] Koushal VK, Goyal V (2015). Patient safety is the need of the hour: a study in nursing department of a tertiary care teaching hospital. Int J Res Foundation Hosp Healthc Admin.

[37] Wami SD, Demssie AF, Wassie MM, Ahmed AN (2016). Patient safety culture and associated factors: a quantitative and qualitative study of healthcare workers’ view in Jimma zone Hospitals Southwest Ethiopia. BMC Health Serv Res.

[38] Khater WA, Akhu-Zaheya LM, Al-Mahasneh SI, Khater R (2015). Nurses' perceptions of patient safety culture in J ordanian hospitals. Int Nurs Rev.

